# Environmental Microbiome of *Tyrophagus Putrescentiae* Culture and Its Changes in Manipulative Experiments

**DOI:** 10.1111/1758-2229.70142

**Published:** 2025-08-01

**Authors:** Jan Hubert, Bruno Sopko, Eliza Głowska‐Patyniak

**Affiliations:** ^1^ Czech Agrifood Research Center – CARC Prague, Prague 6 ‐ Ruzyne Czechia; ^2^ Department of Animal Morphology Faculty of Biology, Adam Mickiewicz University in Poznan Poznan Poland

**Keywords:** allergens, bacteria, digestion, faeces, interaction

## Abstract

Storage mites consume stored products in interaction with environmental microorganisms, resulting in the destruction of infested food and providing specific odours. Here we simulated the effect of mite grazing on oat flakes. Spent growth medium (SPGM) was obtained from seven mite cultures and mixed with oat flakes as the source of faeces and microbes. SPGM‐treated diets were offered to 4 mite cultures. The microbiomes were analysed using sequencing of V4_16S_DNA. Mite growth tests, food preferences, and microbiome changes were observed in correlation with SPGM type and mite cultures. The microbiome consisted of 41 OTUs belonging to mite‐associated bacteria and faeces bacteria. The composition of the microbiome depends more on the source of SPGM than on mite culture. The SPGM diet accelerated mite population growth and influenced mite food choice, although the effect was dependent on both types of SPGM and mite culture. *Kocuria*, *Brevibacterium*, *Virgibacillus*, and *Staphylococcus* profiles in SPGM added into diets showed positive correlations to mite population growth. The *Kocuria* profile in the bodies of mites was positively correlated with mite population growth. The results showed that mites are influenced by SPGM‐treated diets, and mite feeding influences the environmental microbiome. The most beneficial was the mite interaction with *Kocuria*.

## Graphical abstract

Spent growth medium (SPGM) was obtained from seven mite cultures and mixed with oat flakes as the source of faeces and microbes. SPGM‐treated diets were provided to four mite cultures, resulting in accelerated mite population growth and altered mite food preferences.

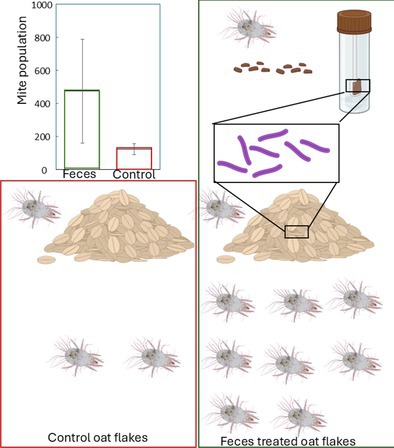

## 1 Introduction

Stored product mites (Acari: Astigmata) are pests of stored commodities for human or animal consumption, such as grain and grain products, cheese, dried ham and sausage, and dog kernels (Canfield and Wrenn 2010; Thind and Clarke 2001; Zhang, Hendrix, et al. 2018). Mites have a short life cycle of 10 days in suitable conditions (Sanchez‐Ramos and Castanera 2001) and multiply almost according to exponential population growth (Aspaly et al. 2007). Without human control, mite feeding completely disintegrates the infested commodity (Robertson 1946; Rybanska et al. 2016). The mite infestation of the stored grain had a typical odour e.g., tridecane or microbial associates 3‐methyl‐1‐butanol, 3‐octanone, and 1‐octen‐3‐ol in the grain (Tuma et al. 1989, 1990). Also, the ham infested by mites provides a smell suggested to be associated with mite‐carrying microbiota (Garcia 2004). It means that mites are living inside microbial‐rich habitats, burrowing tunnels inside the infected food and filling them with faeces, diet debris, and dead bodies. The above‐mentioned observation indicates that mites are surrounded by microorganisms growing in the mite faeces, diet debris, and dead bodies. Among stored product mite species, *Tyrophagus putrescentiae* belongs to the most abundant and frequent species along with the next species *Acarus siro* and *Lepidoglyphus destructor* (Palyvos et al. 2008).

Microorganisms are suggested as a part of mite diets in human environments (Erban and Hubert 2008). Recent studies identified a few bacterial taxa associated with *T. putrescentiae*, i.e., intracellular symbionts: *Cardinium*, *Wolbachia*, gut symbionts: *Bartonella*‐like, *Sodalis*‐like and *Solitalea*‐like bacteria, and *Blattabacterium‐*like putative symbiont (Erban, Klimov, et al. 2016; Erban et al. 2017; Hubert et al. 2012, 2021; Kopecky et al. 2014). The previous experiment showed that the mite microbiome is stable and resistant to environmental stress and nutrient switch, starvation, and faeces addition into the mite diet in terms of acquisition of new bacteria, although there were changes in the microbial profiles. The previous analysis of the effect of diet on *T. putrescentiae* bodies microbiome revealed: (i) mite growth is not correlated with microbiome composition; (ii) microbiomes had a significant impact on mite fitness; and (iii) microbiome changes its profile (*Bacillus*, *Bartonella*‐like, *Solitalea*‐like, *Kocuria*, and *Sodalis*‐like) after diet switch (Hubert, Nesvorna, Sopko, and Green 2023). Expecting mite body‐associated microbiome, there are microbiomes associated with the mite faeces and their environment. For such bacteria, we adopted the term environmental microbiome (Molva et al. 2019, 2020; Nesvorna et al. 2021). The environmental bacteria included *Kocuria*, *Bacillus*, *Staphylococcus*, and the next few abundant taxa (Hubert et al. 2012). The chamber microbiome showed changes in its profile during mite cultivation (Eraso, Guisantes, et al. 1997; Eraso, Martinez, et al. 1997; Eraso et al. 1998; Nesvorna et al. 2021). Under laboratory experiments, feeding of mites on the fungal cultures resulted in the complete destruction of mycelium by the participation of mite grazing and associated microorganisms (e.g., *Alcaligenes faecalis* , *Agrobacterium* sp., *Serratia marcescens* , *Achromobacter* sp.) (Smrz, 1991, 2003; Smrz and Catska 1989). For example, *Bacillus cereus* group was found to be associated with *T. putrescentiae* feeding on dog kernels. The addition of isolated bacteria to the mite diet decreases the growth of mites (Erban, Rybanska, et al. 2016). The observation showed that the faeces microbiome is important to be responsible for the changing of the mite environment and influences mite growth. No microorganisms with pathogenic effects were identified in the analysed environmental microbiomes up to the present time (Hubert et al. 2019; Molva et al. 2019, 2020).

In this study, we sought interactions among members of the environmental microbiome and mites. We expected that bacteria in the environmental microbiome would influence the growth of mites, their food choice, and the composition of their body‐associated microbiome. The contribution of bacteria in mite faeces to the formation and composition of the environmental microbiome in the rearing chambers was analysed. Spent growth medium (SPGM) was used as a source of faeces. SPGM is a sieved fraction after mite cultivation without living mites and eggs. In medical practice, SPGM is used for mite faeces‐associated allergen analyses (Tovey et al. 1981). SPGM was obtained in 7 different *T*. *putrescentiae* cultures added to the mite diet and offered to four *T. putrescentiae* cultures. Mite food choice, population growth, and response to SPGM were correlated with microbial profiles to identify key bacteria within the environmental microbiome.

## 2 Materials and Methods

### 2.1 Mites

The seven *Tyrophagus putrescentiae* (Schrank, 1781) cultures were analysed (Table 1). These cultures are maintained in RICP in darkness with controlled humidity (75% RH) and temperature (25°C ± 1°C); the cultivation was described previously (Hubert et al. 2021). The chambers contained 0.1 ± 0.01 g of rearing diet SPMd (powdered mixture of oat flakes, wheat germ, and Mauripan dried yeast (AB Mauri, Balakong, Malaysia) (10:10:1 w/w) and cca 1000 mites. The chambers were kept in eight replicates per mite culture. The cultures were harvested after 21 days of cultivation. The contents of the cell chambers were harvested from all cultures and used for SPGM isolation.

**TABLE 1:** The list of *Tyrophagus putrescentiae* cultures used for analysis and mite culture descriptions.

ID	Population	Collector	Year	Diet	Site	Intracellular symbionts^#1^	Anal.	SPGM
5 K	Koppert	E. Baal	2012	SPMd	Koppert‐rearing facility, Netherlands	no	+	+
5 L	laboratory	E. Zdarkova	1996	SPMd	grain, Bustehrad, Czechia	*Cardinium*	+	+
5 N	dog	J. Hubert	2007	F	food producing factory, St. Louis, Missouri, USA	*Wolbachia*	—	+
5P	Phillips	T. W. Phillips	2014	SPMd	laboratory strain, Manhattan, Kansas, USA	*Wolbachia*	—	+
5Pi	biscuit	M. Nesvorna	2015	SPMd	contamination of stored biscuits, Prague, Czechia	no	—	+
5S	ham	A. Sala	2013	SPMd	food‐producing factory, Cesena, Italy	*Cardinium*	+	+
5TK	Teplice feed	M. Nesvorna	2015	SPMd	horse feed contamination, Teplice, Czechia	no	+	+

*Note:* SPMd – stored product mite diet, F – dog kernel, Purina Pro Plan FOCUS Adult Sensitive Skin & Stomach Salmon & Rice Formula Dry Dog Food, Anal.—culture used in the experiments, SPGM (spent growth medium)—used in the experiments; #1 according to Hubert et al. (2021).

### 2.2 Spent Growth Medium

The mites and eggs were removed from the collected contents of the chambers by sieving (Mesh size 100 μm). A part of SPGM (0.5 g) was used for SPGM diet preparation by mixing with powdered oat flakes (1/1 w/w). SPGM diets were labelled according to their source (Table 1) as 5 K, 5 L, 5 N, 5 P, 5 Pi, 5 S, and 5 Tk. The control was an oat flake diet without any additives. Another part of SPGM (0.1 g) was used for DNA isolation.

### 2.3 Experiments

The growth test: A 70‐mL flask containing 0.05 ± 0.005 g of SPGM or the control diet with 10 unsexed adults was incubated under controlled conditions (75% RH and 25°C ± 1°C) in six replicates per mite culture and experimental diets. The final mite density was counted after 21 days of cultivation. The experiment was terminated by filling the flask with 30 mL Oudemans' solution (70% ethanol—87 mL, acetic acid—8 mL, and glycerol—5 mL) after which the mites (adults/juveniles) were counted under a dissecting microscope.

The food choice test: The colouring of diets was used according to the protocol developed previously (Vackova et al. 2021), i.e., mixing 0.99 g of diets with 0.01 g of dye. Orange G (O7252‐25G, Sigma‐Aldrich was added to the control diet, and Carmin Red (cat. no. C1022‐25G; Sigma‐Aldrich, Saint Louis, MO, USA) to the SPGM enriched diet. Then the diets were placed into 90 mm Petri dishes with moistened plaster and activated charcoal (9:1) downside. The diets (0.01 g) were in two separate circles (10 mm diameter), and approximately 300 mites were added. The dishes were sealed by Parafilm to block mites from escaping. After 24 h, the mites were collected using a hairbrush into 80% ethanol, and the colour of the mite's midgut was observed through their translucent bodies under a Stemi 2000C dissection microscope (Carl Zeiss, Jena, Germany) and recorded. The observed situations were: (i) SPGM‐treated food—red food boli, (ii) orange food contents—oat flakes food boli, and (iii) no food in the guts named as hungry mites. The experimental design included 12 replicates per SPGM diet and mite culture.

Microbiome sampling: The experimental design followed that of the growth test, with the number of replicates ranging from 6 to 12 per mite culture and diet. The experiment was terminated by filling the culture chamber with 30 mL of PBST (3.2 mM Na_2_HPO_4_, 0.5 mM KH_2_PO_4_, 1.3 mM KCl, 135 mM NaCl) with 0.05% (v/v) Tween 20 detergent (Sigma‐Aldrich, Saint Louis, MO, USA).

### 2.4 DNA Extraction

The PBST extract from SPGM and the contents of the experimental chambers were used for microbiome analyses. The PBST extract was shaken, and 1 mL was transferred into 2 mL APEX ScrewCap Microcentrifuge Tubes (Cat. No. CP5912, Alpha Laboratories, Eastleigh, UK). The tubes were filled with 200 mg 0.3‐mm‐dia. (Cat. No. 11079103gar, BioSpec) and 200 mg 1.0‐mm‐dia. (Cat. No 11079110gar, BioSpec) garnet sharp particles, and 2.85–3.45 mm glass beads (Cat. No. R155761, Carl Roth) and contained 300 μL of extraction buffer from a DNA isolation kit (Cat. No. A2365, Promega) (Hubert, Nesvorna, Bostlova, et al. 2023). Homogenisation was performed using a mini‐BeadBeater 16 (BioSpec Products, Bartlesville, OK, USA). The samples were homogenised for 5 min, and then the samples were centrifuged for 10 min at 6000 g at 4°C before the supernatant was collected. The supernatant was mixed with 20 μL of proteinase K (20 mg/mL) and incubated at 56°C for 1 h. The lysate was then used for DNA isolation using a Wizard SV Genomic DNA Purification System (Cat. No. A2365, Promega) according to the manufacturer's instructions. DNA was eluted in 100 μL of elution buffer.

### 2.5 Microbial Community Characterisation Using 16S rRNA Gene Amplicon Sequencing

Genomic DNA was PCR amplified using a two‐stage “targeted amplicon sequencing (TAS)” protocol as described previously (Caporaso et al. 2012; Hubert et al. 2021; Naqib et al. 2018; Sakai et al. 2004) in the Genome Research Core, Research Resources Center, University of Illinois (Chicago, IL, USA) on a MiniSeq platform (Illumina, San Diego, CA, USA) and employing paired‐end 2 × 153 bp reads. Raw sequences are available at the GenBank SRA, as the project PRJNA800277. The sequence processing was the same as described previously (Hubert et al. 2021), Forward and reverse sequences were aligned and processed using a combination of MOTHUR 1.42.0 (Kozich et al. 2013; Schloss et al. 2009) and UPARSE 11 (Edgar 2013, 2016) according to a previously used protocol (Hubert et al. 2019). The reads were merged, then quality filtered in MOTHUR, and then processed in UPARSE. Sequences were aligned to the mothur‐formatted RDP (Ribosome database project) reference dataset (Cole et al. 2014); and chimeras, ambiguous sequences, chloroplast, and mitochondrial DNA were discarded. The obtained fasta file was used for OTUs (operational taxonomy units at 97% similarity threshold) using RDP reference dataset in UPARSE. After cutting off of low abundant OTUs (fewer than 1000 total reads cca 0.05% of OTU from the dataset), 41 OTUs remained. The sequences were compared to those in GenBank using BLASTn (Altschul et al. 1990), and the closet hits were selected. The identification of *Wolbachia*, *Cardinium*, *Bartonella*‐like, *Blattabacterium*‐like, *Sodalis*‐like, and *Solitalea*‐like OTUs was based on nearly full‐length 16S RNA generated previously (Erban, Klimov, et al. 2016; Kopecky et al. 2014). Then the OTUs sequences were aligned with their closest hit using MUSCLE (Edgar 2004) in SeaView (Galtier et al. 1996), trimmed and exported to MEGA (Kumar et al. 2018), and evolutionary analysis by maximum likelihood method was done using Jukes‐Cantor model (Jukes and Cantor 1969). The final version of the tree was edited in iTOOLv.6 (Letunic and Bork 2024).

### 2.6 Data Analyses

R software v 4.4.1 (R Development Core Team 2024) was applied using Vegan (Oksanen 2024; Zeleny 2021) and WRS2 (Mair et al. 2024), R‐companion (Mangiafico 2016) packages to analyse data. The effect of SPGM‐treated diets and mite cultures on mite population growth was evaluated by trimmed ANOVA. Generalised linear models with binomial error structure (GLM‐b) (Pekar and Brabec 2016) were applied to evaluate the effect of mite culture and SPGM‐treated diets on mite preferences (i.e., feed on faeces or hungry). The effect of tested variables was compared using Bayesian ANOVA in the package Bayes Factor (Morey 2015). The full Bayesian ANOVA/GLM model was executed, followed by an iterative analysis where factors were omitted one by one. The scale of the bars indicates how much worse the model fits when each predictor is omitted (Morey 2015).

The microbiome profile was analysed on standardised data (recalculated to 15,000 reads) using correlation analyses in the Vegan package (Oksanen 2024; Zeleny 2021), i.e., distance‐based redundancy analyses (dbRDA). The first model was constructed to evaluate the effect of mite culture and the source of SPGM‐treated diet on the microbiome using Bray–Curtis distance with the capscale function in Bray–Curtis distance. The next analyses were run separately for mite cultures. As environmental variables, we tested (i) the SPGM origin (mite cultures: 5 K, 5 L, 5 N, 5P, 5Pi, 5S, and 5Tk and control oat flakes), (ii) median values of mite population growth and the proportion of mites on the faeces and hungry mites; (iii) the same factors as ii and microbial profiles of SPGM from mite cultures. The forward selection using the ordistep function in Vegan (Oksanen 2024; Zeleny 2021) was used for the last analyses. The OTUs abundances were compared using SIMPER analyses in the Vegan package, and LOG2 fold difference from control treatments was calculated.

Spearman correlations with permutation‐based P‐values were computed to evaluate the interactions between tested variables and analyzed OTUs, utilizing the median dataset in PAST version 4 (Hammer 2020). The complex heatmap package (Gu 2022a, 2022b; Gu et al. 2016) was used for the visualisation of the interactions.

## 3 Results

### 3.1 Microbiome Description

**FIGURE 1:**
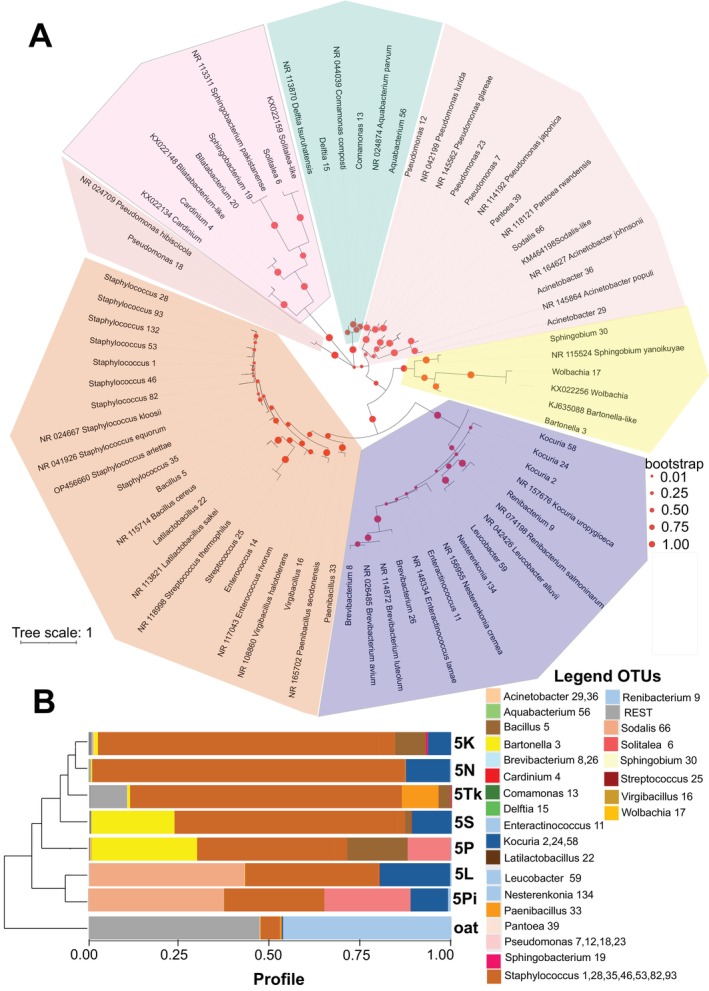
*Tyrophagus putrescentiae* microbiome; A—OTUs based on V4_16S DNA analyses using the Maximum Likelihood method and Jukes–Cantor model (Jukes and Cantor 1969). The selected sequences included 41 OTUs and their closest match in GenBank. The tree with the highest log likelihood (−2617.10) is shown. The branch support was based on 500 bootstraps; the tree was unrooted. The taxonomic position is indicated by colour; B—bacterial profile OTUs in the samples of SPGM originated from 7 mite cultures and rearing diet. SPGM profiles were clustered based on paired group algorithms (UPGMA) in Bray–Curtis distance. These samples were added into mite diets and analysed as SPGM treatment.

The environmental microbiome from all samples was formed by 41 OTUs (Figures 1 and S1, Tables S1–S2). The microbiome was composed of mite‐associated bacteria (i.e., intracellular symbionts: *Cardinium*_4; *Wolbachia*_17, gut symbionts: *Bartonella*‐3, *Sodalis*_66, and *Solitalea*_6, next putative symbiont identified is *Blattabacterium*‐like 20 described previously from *T. putrescentiae* (Erban, Klimov, et al. 2016; Erban et al. 2017; Hubert et al. 2012, 2021; Kopecky et al. 2014). The next OTUs include Firmicutes: *Bacillus*_5, *Staphylococcus*_1,28,35,46,53,82,93, and 132, *Latilactobacillus*_22, *Streptococcus*_25, *Enterociccus*_14, *Virgibacillus*_16, *Paenibacillus*_33; Actinomycetota: *Kocuria*_2,24 and 58, *Renibacterium*_9, *Leucobacter*_59, *Nesterenkonia*_134, *Enteractionococcus*_11, and *Brevibacterium*_8, 28; Alphaproteobacteria: *Spingobium*_30, Gammaproteobacteria: *Pseudomonas*_7,12,18; and 23, *Pantoea*_39, *Acinetobacter*_26, 36; Betaproteobacteria: *Delftia*_15, *Comamonas*_13, and *Aquabacterium*_56; Bacteroidota: *Sphingobacterium*_19.

The SPGM microbiome obtained from initial mite culture media samples differed among mite cultures (5 K, 5 L, 5 N, 5P, 5Pi, 5S, and 5Tk) and from oat flakes (control) (Figure 1B). The difference caused *Leucobacter*_59 to prevail in oat flakes diet, while SPGM microbiomes were formed from *Staphylococcus*_1 as the most abundant bacteria followed by the less abundant mite gut symbiont *Sodalis*_66 (5Pi and 5 L) and *Bartonella*_3 (5S and 5P). The next OTUs were *Kocuria*_2 (5 K, 5Pi, 5S, 5 L, and 5 N), *Solitalea*_6 symbiont (5Pi and 5P), and *Bacillus*_5 (5 K, 5Tk, and 5P).

### 3.2 Mite Population Growth on SPGM Treated Diets

**FIGURE 2:**
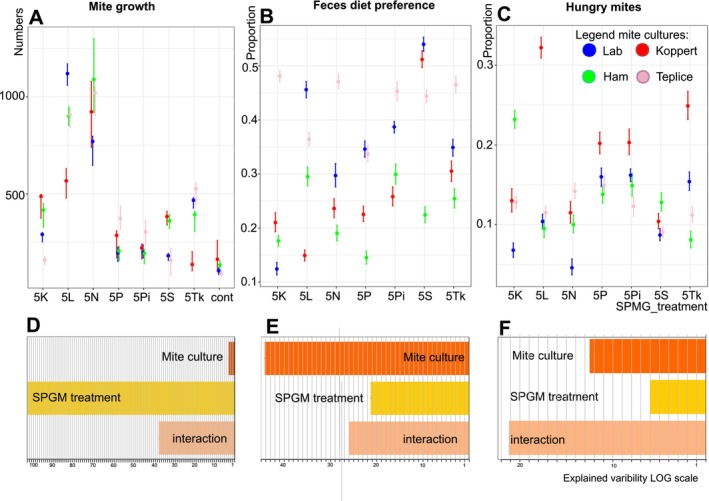
The comparison of *Tyrophagus putrescentiae* population growth (AD) and food choice on SPGM‐treated and control diet (oat flakes) (BC and EF). Mite population growth (A), proportion of mites on SPGM‐treated diet (B), and proportion of hunger mite (C), in experiments are shown. The medians are the points, and 95% confidence limits showed as abscissa. Results of Bayesian ANOVA analyses of the mite culture and SPGM treatment and their interaction as factors contributing to mite density (D), faeces preference (E), and hunger mites (F) are shown. The Bayesian analysis shows the importance of the factors and their interactions. If the factor is omitted, and the bar is oriented to the left (negative), the overall model scores worse, and the factor is important for the model. The length of the bar (logarithmic scale) is the measure of the importance. When the bar is oriented to the right, the model scores better and, therefore, the factor is not important.

Mite population growth was influenced by mite culture, type of SPGM diet, and their interaction (t2way ANOVA: culture F = 17.92, *p* = 0.007; SPGM diet F = 1985, *p* = 0.001, interaction F = 1191, *p* = 0.001). In all cultures, the growth of mites on oat flakes as the control diet was lower, and it was accelerated on the SPGM‐treated diets (Figure 2A). The Bayes ANOVA identified SPGM treatment as the most important factor in terms of explained variability of mite population growth (Figure 2D). The highest growth was on the SPGM diet obtained from cultures 5 L and 5 N when the growth was up to 11 times higher than on oat flakes. The most accelerated growth was in Teplice culture, followed by Koppert, Laboratory, and Ham cultures.

### 3.3 Mite Food Choice Test

The food choice test was based on the preference of mites to feed on a control oat flake diet or one type of the offered SPGM‐treated diet. Because the colour was added to the diet, mites showed coloured food boli in their translucent bodies. Individuals without any food bolus in their gut were considered to be hungry mites. These individuals occurred in all experiments. The mite preference between control and SPGM‐treated diets showed that more than 54% of mite individuals preferred to feed on the oat flakes diet, while 32% fed on the SPGM‐treated diet, and 14% were without coloured food boli (hungry mites). The mite choice was influenced by mite culture, type of SPGM, and their interaction (GLM: culture Chi_(3,24)_ = 4159;*p* < 0.001; SPGM Chi = 1348, *p* < 0.001; interaction SPGM Chi_)_ = 2811, *p* < 0.001) (Figure 2B). The Bayes ANOVA showed that mite culture was the most important factor for mite food selection, followed by interaction (Figure 2E). Surprisingly for the proportion of hungry mites (Figure 2C), the most important was interaction followed by mite culture (Figure 2F). The highest preferences to SPGM diets showed Teplice culture 43% of mite individuals, followed by Koppert (36%), Ham (27%) and Laboratory culture (22%). The most preferred was the 5S‐treated diet, followed by the 5 K and 5P‐treated diets. The highest proportion of hungry mites was 18% of individuals in Ham culture, followed by Laboratory (13%), Teplice (12%), and Koppert (12%) cultures.

### 3.4 Effect of Mite Culture and SPGM Treatment of Faeces Microbiome

**FIGURE 3:**
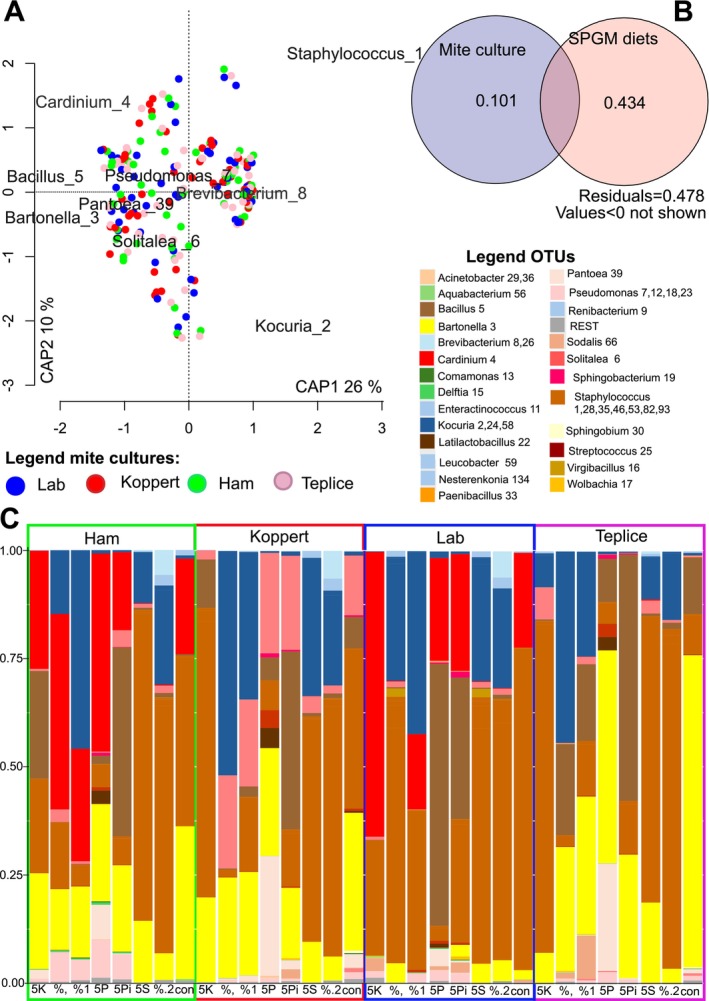
Environmental microbiome of *Tyrophagus putrescentiae* and the effect of mite culture and type of SPGM on microbial profiles.; A—correlation plot showed distribution of samples from mite cultures (Ham, Koppert, Lab, and Teplice) in the first two correlation axes of total dbRDA model and position of bacterial taxa; B—Venn diagram showed explained variability in dbRDA model by mite cultures and SPGM diets; C—profiles of environmental microbiome in mite cultures and on different SPGM treatments. The profiles were calculated from median values per the analysed samples.

In the experiments, environmental microbiome compositions (Figure S1) were influenced by both mite culture and type of SPGM (dbRDA: F(10,209 = 22.881, *p* < 0.001, *R* = 0.523). Type of SPGM (F_(7,209)_ = 24.87, *p* < 0.001, *R* = 0.434) was more important for microbiome composition than mite culture F_(3,209)_ = 18.22, *p* < 0.001, *R* = 0.101) (Figure 3). When the effect of SPGM treatment was analysed separately among mite cultures, the dbRDA models explained 67 to 82% of the total variance in datasets (Table 2). All models for mite cultures (Ham, Koppert, Laboratory, and Teplice) showed separation of samples of chamber microbiome according to SPGM‐added diets. The models showed correlations between SPGM treated by 5 N and 5 L cultures, mite population growth, and faeces preferences in the food choice test.

**TABLE 2:** Correlations between SPGM and chamber microbial profiles in four *Tyrophagus putrescentiae* cultures based on dbRDA models. The models describe the correlation of chamber microbiome profiles to different SPGM added into diets, mite population growth, and food choice in the preference test, and partial models combining bacterial profiles in SPGM or diet and mite population growth and food choice in the preference test. The variables in partial models were selected by forward selection. The models were calculated in Bray–Curtis distances.

Culture	Koppert					Teplice					Lab					Ham				
dbRDA	df		R	F	P	df		R	F	P	df		R	F	P	df		R	F	P
SPGM diet	7	46	0.804	26.91	0.001	7	44	0.820	28.58	0.001	7	51	0.690	16.21	0.001	7	46	0.674	26.91	0.001
Pref	1	50	0.279	7.81	0.001	1	48	0.457	21.78	0.001	1	55	0.256	7.42	0.001	1	51	0.295	10.15	0.001
Nt	1			8.74	0.001	1			11.16	0.001	1			6.88	0.001	1			7.85	0.001
interaction	1			2.80	0.032	1			7.41	0.002	1			4.57	0.005	1			3.35	0.005
partial	6	47	0.797	30.78	0.001	8	43	0.852	30.98	0.001	6	52	0.685	18.87	0.001	7	47	0.675	13.93	0.001

**FIGURE 4:**
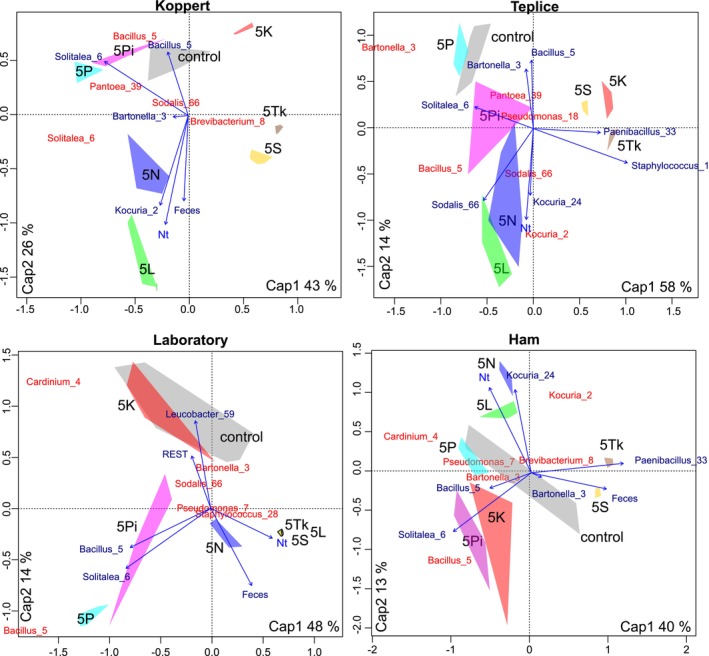
Environmental microbiome of *Tyrophagus putrescentiae* and the effect of mite culture and type of SPGM to microbial profiles based on for dbRDA models for each mite culture separately. The hulls showed the position of samples from the same SPGM‐treated diet. The position of bacterial OTUs in the models are indicated by red colour. The environmental variables correlated to the model are indicated by blue colour. It included the profiles of bacteria in SPGM before treatment and mite preferences to faeces (faeces) in food choice test and mite population density (Nt) in growth test. Before analysis, the mite population density (N) was transformed by dividing by the median values of the growth in the control for each mite culture (Nt). The environmental variables were chosen based on forward selection. Median points for the same SPGM‐treated microbiomes. The correlations between mite population (N) and food choice test (faeces feeding individuals and hungry individuals) are shown.

When SPGM profiles were added as the factor (environmental variable), the presence of *Kocuria*_2 was correlated to the previous environmental variables (Figure 4) and significantly contributed to the composition of the environmental microbiome. This is supported by the Spearman correlation (Table 3). Among bacteria in SPGM, *Kocuria*_2, 24 and 58, *Brevibacterium*_8, 28, *Virgibacillus*_16, and *Staphylococcus*_1, 28, 46 showed positive correlations to mite population growth (Table 3). *Pseudomonas*_12 and *Aquabacterium*_56 showed a negative effect that was observed in population growth. Mite population growth was positively correlated with *Kocuria*_2 in the microbial profile of the chambers. The mites did not show any correlation to bacterial taxa in SPGM in the food choice test. However, the negative correlation between some bacterial taxa (*Kocuria*_24, 58, and *Staphylococcus*_28, 93) and hungry individuals indicates that these taxa should be important for mite food preference.

**TABLE 3:** Spearman correlations among mite *Tyrophagus putrescentiae* population growth, food preference, and chamber microbiome composition. The correlations were calculated to the SPGM composition and to microbiomes. Only coefficients with significant permutational P (*P* ≥ 0.05) are shown. The values were indicated by colour.

Microbiome Factor	SPGM composition				Microbiome composition			
	Nt1	Nt2	Faeces	Hungry	Nt1	Nt2	Faeces	Hungry
*Staphylococcus*_1	0.49	0.00	0.00	0.00	0.00	0.00	0.00	0.00
*Kocuria*_2	0.53	0.52	0.00	0.00	0.49	0.47	0.00	−0.41
*Cardinium*_4	0.00	0.00	0.00	−0.45	0.00	0.00	−0.52	0.42
*Pseudomonas*_7	0.00	0.00	0.00	0.00	0.00	0.00	−0.52	0.43
*Staphylococcus*_28	0.00	0.40	0.00	−0.42	0.00	0.00	0.00	0.00
*Brevibacterium*_8	0.49	0.51	0.00	0.00	0.00	0.00	0.00	0.00
*Leucobacter*_59	−0.42	0.00	0.00	0.00	0.00	−0.44	0.00	0.46
*Enterococcus*_14	0.00	0.00	0.00	0.00	0.00	0.00	0.00	0.00
*Renibacterium*_9	0.35	0.00	0.00	0.00	0.00	0.00	0.42	0.00
*Kocuria*_24	0.53	0.00	0.00	−0.47	0.00	0.00	0.00	−0.40
*Wolbachia*_17	0.00	0.00	0.00	0.00	0.00	0.00	0.00	0.42
*Pseudomonas*_12	−0.53	0.00	0.00	0.00	0.00	0.00	−0.39	0.42
*Comamonas*_13	0.00	0.00	0.00	0.00	0.00	0.00	0.00	0.42
*Virgibacillus*_16	0.49	0.51	0.00	0.00	0.00	0.00	0.00	0.00
*Delftia*_15	0.00	0.00	0.00	0.00	0.00	0.00	−0.45	0.45
*Brevibacterium*_26	0.49	0.51	0.00	0.00	0.00	0.00	0.00	0.00
*Nesterenkonia*_134	0.48	0.00	0.00	−0.45	0.00	0.00	0.00	0.00
*Staphylococcus*_132	0.00	−0.40	0.00	0.00	0.00	0.00	0.00	0.00
*Staphylococcus*_93	0.00	0.00	0.00	−0.46	0.00	0.00	0.00	−0.42
*Aquabacterium*_56	−0.54	0.00	0.00	0.00	0.00	0.00	0.00	0.00
*Staphylococcus*_53	0.39	0.00	0.00	0.00	0.00	0.00	0.00	0.00
*Paenibacillus*_33	0.00	0.00	0.00	0.00	0.00	0.00	−0.40	0.00
*Kocuria*_58	0.50	0.00	0.00	−0.39	0.00	0.00	0.00	0.00
*Sphingobium*_30	0.00	0.00	0.00	0.00	0.00	0.48	0.00	0.00
*Staphylococcus*_46	0.45	0.44	0.00	0.00	0.00	0.00	0.00	0.00
*Streptococcus*_25	0.00	0.00	0.00	0.00	0.00	0.00	−0.42	0.39
REST	0.00	0.00	0.00	0.00	0.00	0.00	−0.40	0.53

*Note:* Nt means transformed numbers of mite (divided by numbers on control in 1 total data set, 2 datasets without control observation, Faeces means proportion of mites on SPGM diet, Hungry means proportion of mites without any food bolus.

**FIGURE 5:**
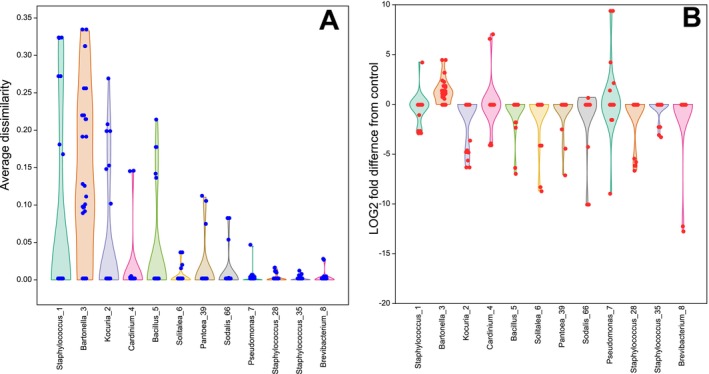
Comparison of relative abundance of main OTUs in the environmental microbiome of *Tyrophagus putrescentiae*; the average dissimilarity (A) and differences from the control (B) are shown. The data were calculated in SIMPER analyses and visualised as violin and jitter plots. For the analyses, only those with significant permutational tests were used (*p* ≤ 0.05), while insignificant cases were replaced by 0. The differences were calculated as LOG2 of relative abundance in the samples divided by control.

The Bayes ANOVA for profile distribution of separate bacterial taxa revealed that SPGM treatment is the most important factor for the relative abundance of the majority of OTUs, i.e., *Staphylococcus*_1, 28, 35, *Bacillus*_5, *Kocuria*_2, *Breavibacterium*_8, and *Pantoea*_39. The mite culture is an important factor for *Pseudomonas*_7 and *Solitalea*_6. *Bartonella*_3 had both these factors of the same importance. Differently, *Sodalis*_66 relative abundance is influenced by the combination of mite culture and SPHM treatment, while the rest of the factors were unimportant (Figure S2). Both *Staphylococcus*_1 and *Bartonella*_3 were identified as key contributors to the dissimilarity among chamber microbiomes, because they showed the highest average dissimilarity values for SPGM treatment × control comparison of microbiomes (Figure 5B). While *Pseudomonas*_7, *Sodalis*_66, and *Brevibacterium*_8 had the highest differences in relative abundance expressed as LOG2 fold difference from the control profile.

## 4 Discussion

The study showed that SPGM diet addition accelerated *Tyrophagus putrescentiae* population growth and influenced its food choice. *Kocuria*, *Brevibacterium*, *Virgibacillus*, and *Staphylococcus* profiles in SPGM added into diets showed positive correlations to mite population growth. It corresponds to previous findings that the environmental microbial community forms pools for bacterial recolonisation of mite hosts (Adair and Douglas 2017). Because host‐associated communities are expected to be formed from microbes in external environments, including those horizontally transmitted with a free‐living phase (Adair and Douglas 2017), bacteria from the environmental microbiome are also found within the mite internal microbiome. In *T. putrescentiae*, it includes gut symbionts and faeces identified previously (Erban, Klimov, et al. 2016; Erban et al. 2017; Hubert et al. 2012, 2021) as faeces associated and those which belonged to the cultivable part of the mite‐associated microbiome (e.g., *Kocuria*, *Staphyloccocus*, and *Bacillus*) (Hubert et al. 2012). However, the low profile of intracellular symbionts (*Wolbachia* and *Cardinium*) was identified, also. It is due to the sampling method because the samples still contain fragments of mite bodies with symbiont DNA. The effect of intracellular bacteria (5 L and 5S) culture on environmental microbiome formation was observed (5 L and 5S) culture similary as we suggested them to influence mite gut microbiome previously (Nesvorna et al. 2020).

The previous study showed that although mite faeces can serve as the vector for faeces‐associated bacteria, no positive effect of faeces‐treated diet on *T. putrescentiae* population growth was observed (Green et al. 2022). Although *Bartonella*‐like symbionts in the mite body microbiome profile increase in the faeces diet‐treated mite, this correlated with an increase in population growth (Hubert et al. 2018). Both these experiments were on the optimal mite‐rearing diet. Therefore, in this study, the suboptimal oat flake diet (Hubert, Nesvorna, Sopko, and Green 2023) was used, and population growth was higher on faeces‐treated diet in all experiments. However, the mites still showed a low preference for faeces‐containing diets, although it did not exclude coprophagy (Onchuru et al. 2018) as a transmission mechanism of microbes in mites. The results showed that all SPGM‐treated diets were more suitable for mite growth than the control.

Differently from mite bodies, the environmental microbiome was more influenced by the microbial source of SPGM treatment than by mite culture. In the previous study analysing the effect of different diets on *T. putrescentiae* cultures and mite body microbiome, we identified (i) *Kocuria* and *Staphylococcus* were influenced by all analysed factors, i.e., mite culture, diet, and their interaction, while *Bacillus* was influenced by mite culture only (Hubert, Nesvorna, Sopko, and Green 2023). Such responses were not observed in this study with the environmental microbiome when SPGM treatment was the most important factor for these bacteria. The interpretation is that SPGM addition provides inoculum of the bacteria into the mite environment and influences further bacteria growth there. It means that SPGM inoculated bacteria into SPGM‐treated diets. There was an interaction with the activity of the mites because an SPGM‐treated diet influences both mite population growth and food choice. However, the correlation between mite population growth and food choice to the composition of the final environmental microbiome means that mite feeding forms an environmental microbiome also. The previous study showed that T. putrescentiae has a digestive enzyme (lysozyme activity) to hydrolyse cell walls of Gram‐positive bacteria (Childs and Bowman 1981); however, the population growth was not accelerated by *Micrococcus lysodeikticus* addition (Erban and Hubert 2008), even if it was suppressed by *B. cereus* addition reduced population growth (Erban, Rybanska, et al. 2016). The food boli formed exclusively or partly from bacteria were observed in *Carpoglyphus latis* (Hubert et al. 2015) but were not identified in *T. putrescentiae* (Erban, Klimov, et al. 2016). Given altogether, the mites are not exclusively feeding on bacteria, although they are able to digest their cell walls. The results of the study showed that SPGM addition to the diet accelerated mite population growth and influenced mite food choice. *Kocuria*, *Brevibacterium*, *Virgibacillus*, and *Staphylococcus* profiles in SPGM added into diets showed positive correlations to mite population growth. *Kocuria* in the environmental microbiome was positively correlated with the mite population growth. The results showed that mites are influenced by SPGM addition to their diets, and mite feeding influences the environmental microbiome.

The alternative hypothesis to explain the positive effect of SPGM‐treated diet on mite growth is an external rumen hypothesis (Nalepa et al. 2001). The “external rumen” hypothesis, proposes that arthropods re‐ingest their faeces to exploit the products of microbial activity (Lavelle 1997). The detritivore‐microbial interactions during coprophagy include gregarious behaviour and faeces or the bacteria in the attract arthropods to feed them and re‐colonise their digestive tract by bacteria (Wada‐Katsumata et al. 2015). Such activity is expected in *T. putrescentiae*, because a part of bacterial community (*Sodalis*‐like, *Solitalea*‐like, and *Bartonella*‐like) is present in mite bodies and faeces, but not eggs. Due to their occurrence, we suggested that these bacteria are gut‐associated and for their transmission coprophagy is a necessary strategy (Nalepa et al. 2001). It opens the suggestion that the environmental microbiome had beneficial effect of these bacteria could lead to the partial hydrolyzes of the diet and the contents of the cell chamber. The bacterial cultivation and enzymatic activity test suggested that mite associated‐bacteria have the ability to hydrolyze and utilise chitin from fungal cell walls (Smrz and Catska 2010). Such bacterial activities continue in the mite faeces. A wide spectrum of bacillolysins was identified in *B. cereus* SPGM and the interaction of *Bacillus* + mite proteases and exochitinase was suggested as a possible degradation of mite or fungal residues in the mite contaminated environments (Erban, Rybanska, et al. 2016). By this hydrolysis activity, the bacteria can reallocate nutrients to the mites. The study showed the most beneficial was the mite interaction with *Kocuria* in the environmental microbiome. *Kocuria* was known to be associated with the bark beetle *Dendroctonus rhizophagus* and *Kocuria* is suggested to relate to nitrogen fixation and cellulose breakdown (Morales‐Jimenez et al. 2012). *Kocuria rosea* produces keratinase enzyme (Bernal et al. 2006), which enables the degradation of keratin as a possible food (skin and nail debris) source of mites in house dust.

However, the mass rearing of mites simulates natural conditions; the limitation of the experiment was that it was in the laboratory, and the microbiome of mites in the home environment is not known to provide a comparison if these interactions occur outside the laboratory. Proteobacteria, Actinobacteria, and Firmicutes were the leading phyla, and *Acinetobacter*, *Paracoccus*, and *Kocuria* were the dominating genera in university dormitory (Wu et al. 2022). Similarly, *Kocuria*, B*lastococcus*, and *Massilia* were the prevailing genera in particulate dust, and *Acinetobacter*, *Lactobacillus*, and *Corynebacterium* were the dominating genera in flocculent fibrous dust (Li et al. 2022). In an experimental porcine skin model microbiome, *Staphylococcus was* abundant in scabies mite infested animals and showed a much reduced presence of *Lactobacillus* compared to the microbiome control animals (Swe et al. 2014). *Kocuria* occurred in natural *Carpoglyphus lactis* cultures (Carbonero‐Pacheco et al. 2023). *Kocuria* was the most frequent isolate bacteria from surface cleaned ticks (Li et al. 2014). In newly emerged buprestid adults (*Agrilus mali*), *Kocuria* were extremely abundant bacteria (Zhang, Jiao, et al. 2018). These studies support the natural occurrence of *Kocuria* in mite cultures and indicate that the observed interaction between mites and environmental bacteria could occur in the field.

During the feeding, the mites contaminate infested food with allergens, which are associated either with their bodies or faeces (Tovey et al. 1981). There are well‐documented allergen diseases associated with the allergen diseases of farmers (Iversen et al. 1990; van Hage‐Hamsten et al. 1985), food industry workers (Tafuro et al. 2015; Tee et al. 1992), but also city populations (Jogi et al. 2020; Vidal et al. 1997) caused by *T. putrescentiae* (Arlian et al. 1997). Recently, more than 40 biochemically different compounds have been described to start allergen reactions. Among these compounds are those associated with controlling mite‐associated bacteria or digestive enzymes responsible for proteins and starch breakdown in diets (Vrtala 2022). The possible weakness of the study is that it was done on laboratory cultures, so field experiments are necessary to confirm environmental bacteria in mite infestation. Our study showed that the environmental microbiome is a product of mite‐feeding activity and faeces. The inhabitants of the microbiome are bacteria, which also occur in the human environment Bacteria are important from the medical point of view due to the connection of mites to allergen production and contamination of food for human consumption and animal feed.

## Author Contributions

**Jan Hubert:** designed and organised experiments; wrote the manuscript. **Eliza Głowska‐Patyniak:** designed and organised experiments; wrote the manuscript. **Bruno Sopko:** provided Bayesian statistical analysis; wrote the manuscript.

## Conflicts of Interest

The authors declare no conflicts of interest.

## Supporting information


**FIGURE S1:** Environmental microbiome of *Tyrophagu*s putrescentiae visualised as heatmap of relative abundance. The medians of relative abundance calculated per SPGM treatments were LOG2 transformed. The columns (OTUs) were clustered. The rows showed SPGM treatments for analysed culture. The median values for mite population growth and proportion of mites in food choice test (Faeces and Hungry) are visualised as bar graphs.

**FIGURE S2:** The analysis of dominant OTUs relative abundance in the environmental microbiome of *Tyrophagus putrescentiae*. Results of Bayesian ANOVA analyses of the mite culture and SPGM treatment and their interaction as factors contributing to OTUs relative abundance. The Bayesian analysis shows the importance of the factors and their interactions. If the factor is omitted, and the bar is oriented to the left (negative), the overall model scores worse and the factor is important for the model. The length of the bar (logarithmic scale) is the measure of the importance. When the bar is oriented to the right, the model scores better and, therefore, the factor is not important.

**TABLE S1:** Environmental microbiome of *Tyrophagus putrescentiae*, the list of identified OTUs and their taxonomical identification.
**TABLE S2:** The standardised dataset of microbial profiles in environmental microbiome of *Tyrophagus putrescentiae*. The samples include SPGM (5 K, 5 L, 5 N, 5P, 5Pi, 5S, 5Tk and control) and microbiome of diets treated by SPGM of four mite cultures (Ham, Koppert, Laboratory, and Teplice).

## Data Availability

The data that supports the findings of this study are available in the supplementary material of this article.
