# Supplementary figures and images for: Environmental Microbiome of Tyrophagus Putrescentiae Culture and Its Changes in Manipulative Experiments
Source: Environ Microbiol Rep. 2025 Aug 1;17(4):e70142. doi: 10.1111/1758-2229.70142 (PMC12314310; doi:10.1111/1758-2229.70142)

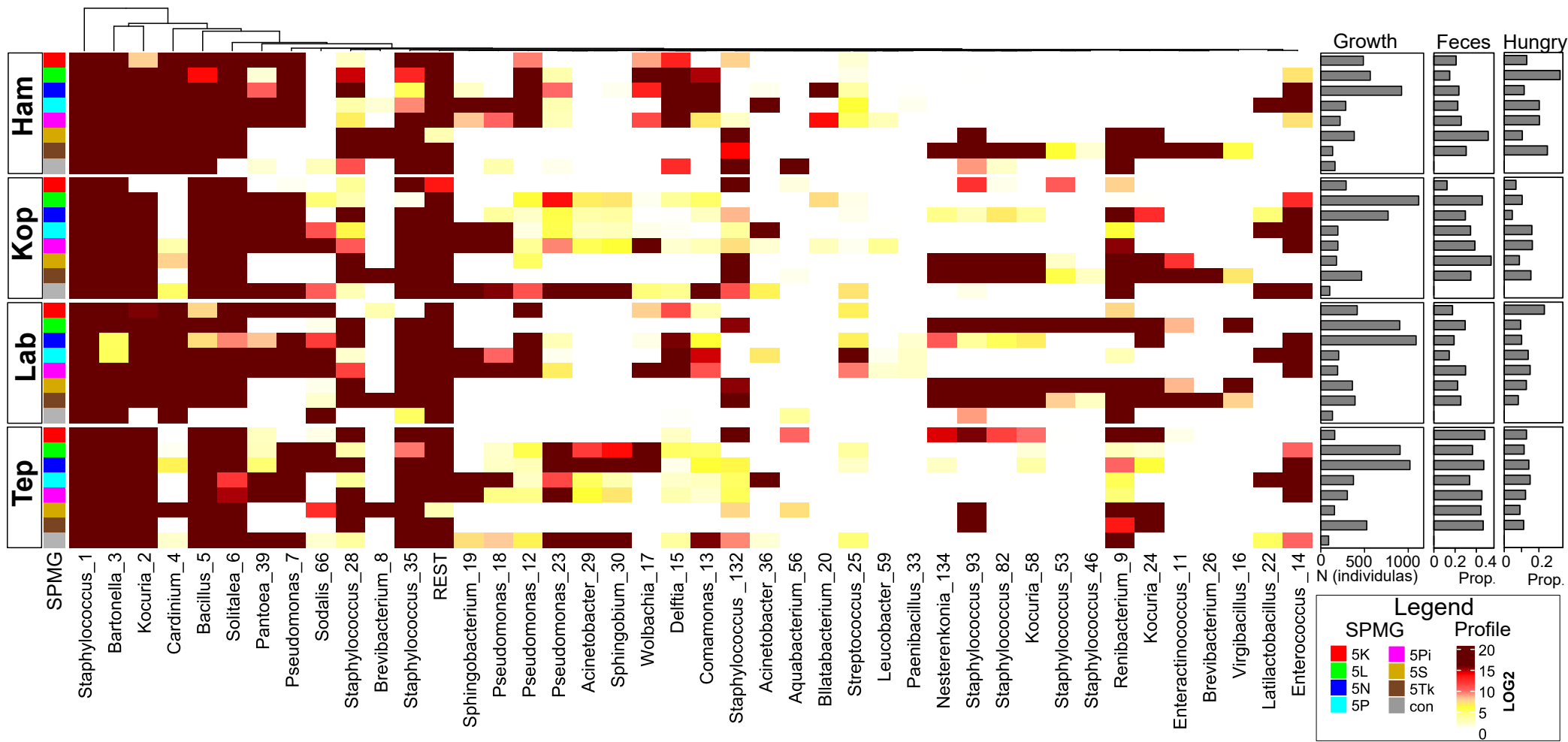

Supplement: Supplementary file 1 — FIGURE S1: Environmental microbiome of Tyrophagus putrescentiae visualised as heatmap of relative abundance. The medians of relative abundance calculated per SPGM treatments were LOG2 transformed. The columns (OTUs) were clustered. The rows showed SPGM treatments for analysed culture. The median values for mite population growth and proportion of mites in food choice test (Faeces and Hungry) are visualised as bar graphs. [file EMI4-17-e70142-s003.pdf]

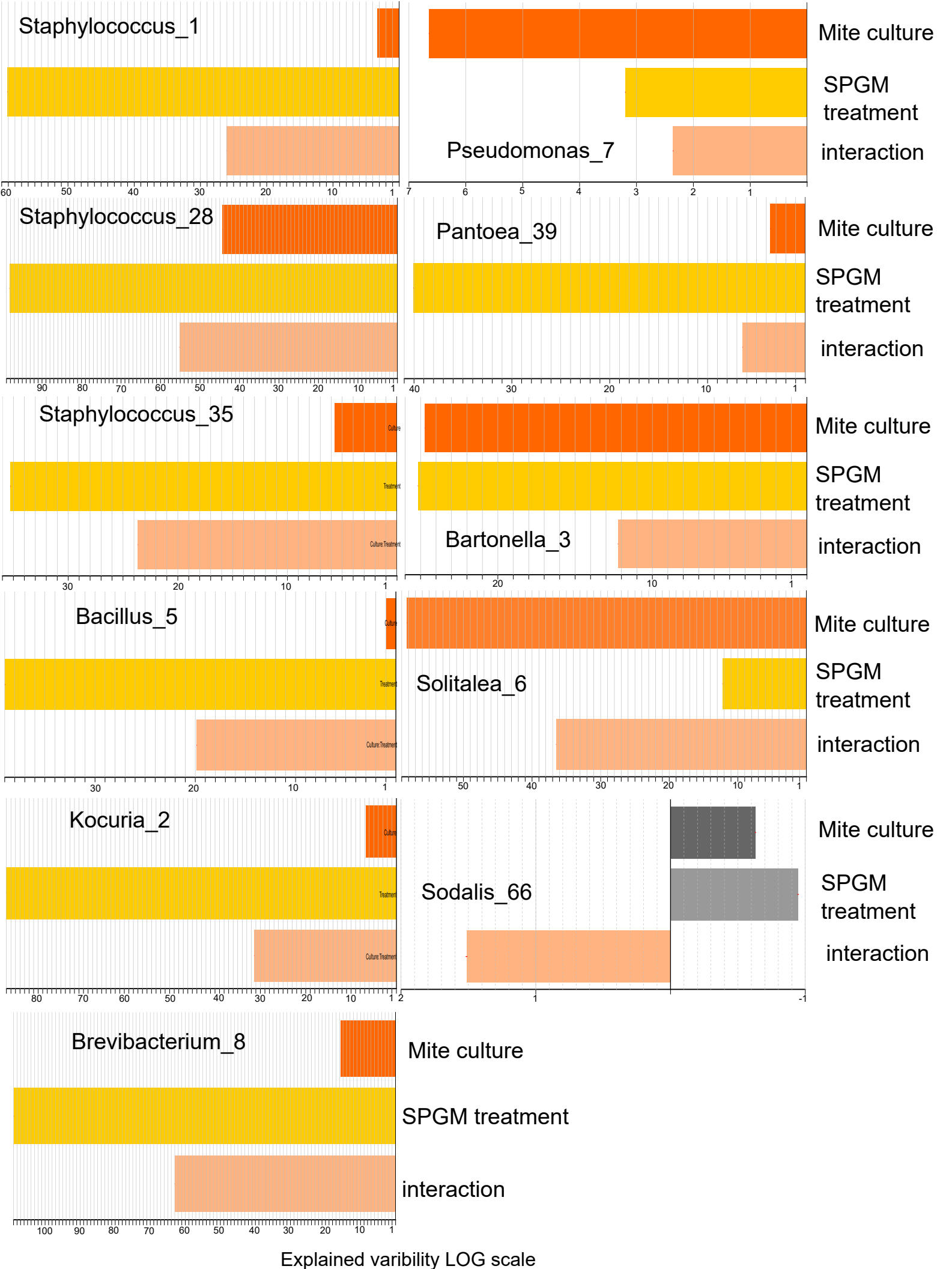

Supplement: Supplementary file 2 — FIGURE S2: The analysis of dominant OTUs relative abundance in the environmental microbiome of Tyrophagus putrescentiae. Results of Bayesian ANOVA analyses of the mite culture and SPGM treatment and their interaction as factors contributing to OTUs relative abundance. The Bayesian analysis shows the importance of the factors and their interactions. If the factor is omitted, and the bar is oriented to the left (negative), the overall model scores worse and the factor is important for the model. The length of the bar (logarithmic scale) is the measure of the importance. When the bar is oriented to the right, the model scores better and, therefore, the factor is not important. [file EMI4-17-e70142-s001.pdf]
